# Multiple pathways for the formation of the γ-glutamyl peptides γ-glutamyl-valine and γ- glutamyl-valyl-glycine in *Saccharomyces cerevisiae*

**DOI:** 10.1371/journal.pone.0216622

**Published:** 2019-05-09

**Authors:** Olga A. Sofyanovich, Hiroaki Nishiuchi, Kazuo Yamagishi, Elena V. Matrosova, Vsevolod A. Serebrianyi

**Affiliations:** 1 Ajinomoto-Genetika Research Institute, Moscow, Russian Federation; 2 Process Development Laboratories, Research Institute for Bioscience Products & Fine Chemicals, Ajinomoto Co., Inc, Kawasaki, Kanagawa, Japan; Universite Paris-Sud, FRANCE

## Abstract

The role of glutathione (GSH) in eukaryotic cells is well known. The biosynthesis of this γ-glutamine tripeptide is well studied. However, other γ-glutamyl peptides were found in various sources, and the pathways of their formation were not always clear. The aim of the present study was to determine whether *Saccharomyces cerevisiae* can produce γ-glutamyl tripeptides other than GSH and to identify the pathways associated with the formation of these peptides. The tripeptide γ-Glu-Val-Gly (γ-EVG) was used as a model. Wild-type yeast cells were shown to produce this peptide during cultivation in minimal synthetic medium. Two different biosynthetic pathways for this peptide were identified. The first pathway consisted of two steps. In the first step, γ-Glu-Val (γ-EV) was produced from glutamate and valine by the glutamate-cysteine ligase (GCL) Gsh1p or by the transfer of the γ-glutamyl group from GSH to valine by the γ-glutamyltransferase (GGT) Ecm38p or by the (Dug2p-Dug3p)_2_ complex. In the next step, γ-EV was combined with glycine by the glutathione synthetase (GS) Gsh2p. The second pathway consisted of transfer of the γ-glutamyl residue from GSH to the dipeptide Val-Gly (VG). This reaction was carried out mainly by the (Dug2p-Dug3p)_2_ complex, whereas the GGT Ecm38p did not participate in this reaction. The contribution of each of these two pathways to the intracellular pool of γ-EVG was dependent on cultivation conditions. In this work, we also found that Dug1p, previously identified as a Cys-Gly dipeptidase, played an essential role in the hydrolysis of the dipeptide VG in yeast cells. It was also demonstrated that γ-EV and γ-EVG could be effectively imported from the medium and that γ-EVG was imported by Opt1p, known to be a GSH importer. Our results demonstrated that γ-glutamyl peptides, particularly γ-EVG, are produced in yeast as products of several physiologically important reactions and are therefore natural components of yeast cells.

## Introduction

**Fig 1:**
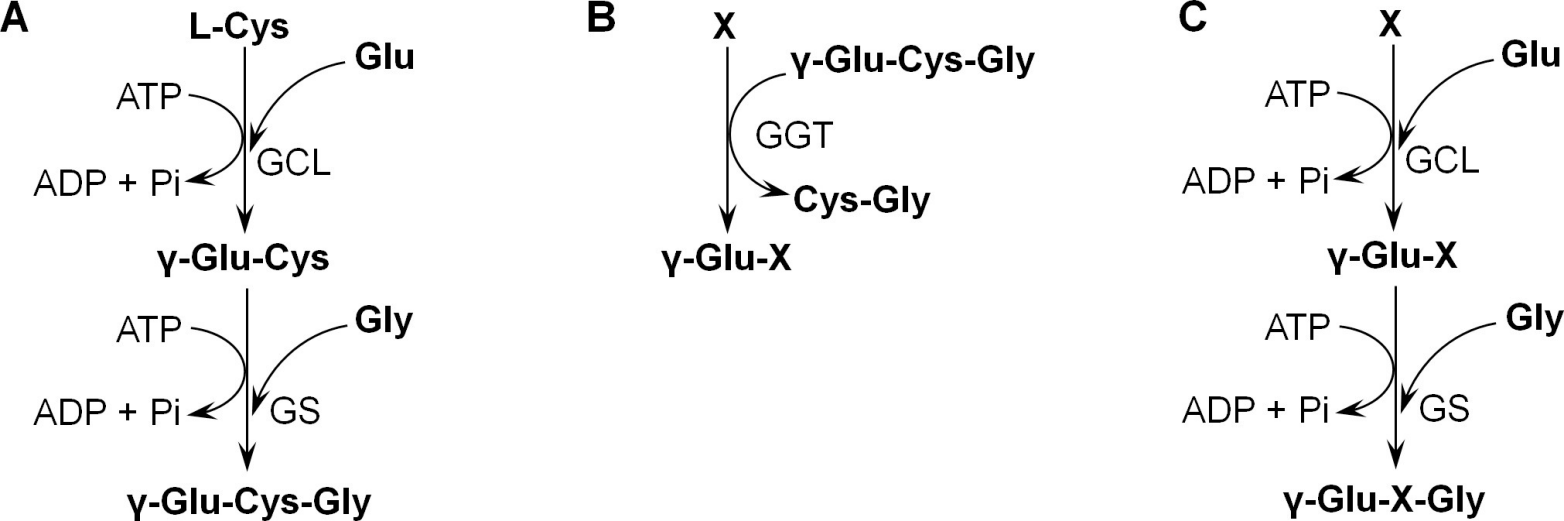
Glutathione biosynthesis pathway and two possible pathways for the synthesis of γ-glutamyl peptides. **A. Schematic depiction of glutathione biosynthesis.** GCL, glutamate-cysteine ligase; GS, glutathione synthetase. **B. Synthesis of γ-glutamyl peptides during glutathione degradation.** γ-Glutamyltransferase (GGT) transfers γ-glutamyl residues from GSH to amino acids or peptides (X). **C. Synthesis of γ-glutamyl peptides as a byproduct of GSH biosynthesis.** GCL uses another amino acid (X) instead of cysteine as a substrate and produces a γ-glutamyl dipeptide (γ-Glu-X). Then, GS uses this peptide as a substrate and produces a tripeptide (γ-Glu-X-Gly).

The most well-known γ-glutamyl compound is the tripeptide γ-L-glutamyl-L-cysteinylglycine, also known as glutathione (γ-Glu-Cys-Gly, GSH). GSH plays an important role in many physiological processes, including maintenance of redox balance and detoxification of cells. The GSH biosynthetic pathway has been well studied in many organisms; this pathway comprises two steps (Fig 1A). In the first step, glutamate-cysteine ligase (γ-glutamylcysteine synthetase, GCL, EC 6.3.2.2) produces γ-glutamylcysteine (γ-GC) from glutamate and cysteine. In the second step, γ-GC is combined with glycine by glutathione synthetase (GS, EC 6.3.2.3) [1, 2]. In some organisms, these two reactions are carried out by a single enzyme [3]. A recent study also suggested the potential importance of the GSH precursor γ-GC in physiological activities [4–6]. In addition to GSH and γ-GC, several other γ-glutamyl compounds, mainly dipeptides, have been identified from many sources [7–23]. The roles of these compounds in cells is not always clear, but some have been detected in important mammalian tissues, such as the brain [11] and eyes [23], which may indicate the involvement of these compounds in signaling pathways. This hypothesis is supported by the fact that theanine (γ-glutamylethylamine), a substance that was first identified in tea leaves [24], has a well-known stimulatory effect [25]. Recently, there has been an interest in using γ-glutamyl peptides for the treatment of Alzheimer’s disease [26, 27].

While medical studies on γ-glutamyl peptides have started relatively recently, there is another area in which the role of these compounds has been well studied. Some of these compounds are important (or even principal) components of the tastes and smells of food. For example, γ-Glu-S-allyl-Cys was found to be one of the compounds that determines the taste properties of garlic [18]; γ-Glu-S-propenyl-Cys sulfoxide is associated with the taste of onion [19]; γ-Glu-Leu, γ-L-Glu-Val, and γ-Glu-Cys-β-Ala determine the taste properties of beans [20]; and γ-Glu-Phe, γ-Glu-Tyr, γ-Glu-Leu, γ-Glu-Glu, γ-Glu-Gly, γ-Glu-Gln, γ-Glu-Met and γ-Glu-His are key components that influence the flavor of mature cheese [21, 22]. Moreover, some data suggest that GSH is associated with flavor formation in several foodstuffs, including meat [28]. Notably, a significant amount of γ-GC has been detected in some foodstuffs, such as chicken gizzard and yeast [29]. In addition to their own taste, several γ-glutamyl compounds enhance the taste of food (continuity, richness and thickness). Compounds that possess these properties have been coined “kokumi compounds” or “kokumi flavor compounds” [18–20, 28].

Although GSH biosynthesis has been well studied in many organisms, there is little data regarding the mechanism of formation of other γ-glutamyl compounds. For several compounds, two different mechanisms were demonstrated in *in vitro* and *in vivo* experiments. Both of these mechanisms were associated with GSH metabolism. The first mechanism was based on the known pathway of GSH degradation (Fig 1B), which is carried out by γ-glutamyltransferase (GGT, EC 2.3.2.2), previously called γ-glutamyltranspeptidase. This enzyme transfers the γ-glutamyl moiety from GSH to any amino acid or water [30, 31]. Several γ-glutamyl compounds have been produced by using this enzyme *in vitro* [30–32]. Moreover, two γ-glutamyl dipeptides, namely, γ-Glu-Glu and γ-Glu-Gly, were identified in the yeast *Saccharomyces cerevisiae*, and there was some evidence that these compounds were synthesized by GGT [33]. It has also been proposed that γ-glutamyl peptides found in cheese are synthesized by GGT produced by the fungus *Penicillium roqueforti* [34].

The second mechanism involved the relatively broad substrate specificity of the GSH biosynthetic enzymes (Fig 1C). It has been demonstrated *in vitro* for several studied GCLs that these enzymes can bind glutamate with some other amino acids besides cysteine, producing the corresponding γ-glutamyl dipeptides. In turn, GS can recognize these dipeptides and combine them with glycine [35–41]. In particular, it was demonstrated that ophthalmic acid (γ-L-glutamyl-L-α-amino-n-butyrylglycine, γ-Glu-Abu-Gly), a tripeptide primarily isolated from calf lenses, is synthesized via this pathway [23].

It can also be presumed that γ-glutamyl peptides can be formed as a byproduct in other reactions in which γ-glutamyl phosphate is an intermediate. For example, glutamine synthetase and γ-glutamylmethylamide synthetase were used for *in vitro* synthesis of theanine from glutamate, ethylamine and ATP [42, 43]. In this regard, it is worth mentioning that accumulation of γ-glutamyl phosphate due to mutation in the proline biosynthesis pathway suppressed the GSH auxotrophy of *S*. *cerevisiae gsh1*Δ strain [44]. Moreover, γ-glutamyl kinase Pro1p was used for improving the GSH yield in the GSH-producing *S*. *cerevisiae* strain [45]. These experiments indicated that γ-glutamyl kinase also possesses relatively broad substrate specificity.

Wild-type *S*. *cerevisiae* strains synthesize approximately 10 μmol (0.3 mg) GSH per gram dry cell weight (DCW) when cultivated in a minimal synthetic medium, which corresponds to an intracellular concentration of 4 mM [46, 33]. The synthesis of GSG in *S*. *cerevisiae* is catalyzed by GCL and GS, which are encoded by *GSH1* and *GSH2*, respectively [47–51]. In addition, *S*. *cerevisiae* synthesizes GGT, which is encoded by *ECM38* [52–54]. Thus, it is very likely that *S*. *cerevisiae* can produce γ-glutamyl compounds via one of the above described mechanisms associated with GSH metabolism. Moreover, several γ-glutamyl di- and tripeptides were recently identified in yeast extract [55], and some experimental data indicate that in *S*. *cerevisiae*, GGT produces two γ-glutamyl peptides, namely, γ-Glu-Glu and γ-Glu-Gly [33].

In the present study, we have chosen the γ-glutamyl peptide γ-L-glutamyl-L-valyl-glycine (γ-Glu-Val-Gly, γ-EVG) as a model to determine whether *S*. *cerevisiae* can produce γ-glutamyl tripeptides other than GSH. We chose this particular peptide because it was recently discovered that it possesses strong kokumi properties [56] and it has been detected in several traditional foods and in beer [13–17]. In addition, *S*. *cerevisiae* is used for the production of yeast extract, which is widely used as a food ingredient. Therefore, the ability of *S*. *cerevisiae* to produce this peptide would be interesting from a biotechnological perspective. In this study, we detected γ-EVG in yeast extract and demonstrated that γ-EVG may be produced in yeast via two pathways: (i) by GCL and GC using glutamate, valine and glycine as precursors; or (ii) via transfer of the γ-glutamyl residue from GSH to the dipeptide Val-Gly (VG). This reaction is carried out mainly by the (Dug2p-Dug3p)_2_ complex, which mediates an alternative GSH degradation pathway [57–59], whereas GGT Ecm38p does not participate in this reaction or its contribution is negligible. We also demonstrated that the dipeptide γ-EV may be produced via transfer of the γ-glutamyl group from GSH to valine. This reaction can be carried out by Ecm38p or by the (Dug2p-Dug3p)_2_ complex. In addition, we found that Dug1p, previously identified as Cys-Gly dipeptidase, possesses high activity toward the dipeptide VG and demonstrated that γ-EVG can be effectively imported from the medium by Opt1p (Hgt1p), a known importer of GSH.

## Materials and methods

### Materials, culture media and yeast strains

Media components were purchased from BD (Difco), USA. Amino acids (LAA21 set) were purchased from Sigma-Aldrich Chemie GmbH Munich, Germany. Uracil was purchased from AppliChem GmbH, Germany. Geneticin (G418) and phleomycin were purchased from InvivoGen, USA. γ-EV and VG were purchased from Bachem AG, Switzerland, and γ-EVG was synthesized by Kokusan Chemical Co., Ltd., Japan. Culture media preparation and yeast strain manipulation were carried out according to standard procedures [60]. For transformation of yeast cells, the lithium acetate method was used [61]. The culture media used in this work are listed in Table 1. For selection for geneticin or phleomycin resistance after yeast transformation, YPD agar plates were supplemented with 200 mg/L geneticin or 7.5 mg/L phleomycin, respectively. For selection of the Ura^+^ phenotype, SD agar plates were used. For cultivation of Ura^-^ strains, the synthetic media were supplemented with 20 mg/L uracil. The yeast strains used in this study were derivatives of S288C. The construction of these strains is described in S1 File. The MATa yeast deletion collection (95401.H2) was purchased from Invitrogen Life Technologies Ltd., Carlsbad, USA.

**Table 1:** Culture media used in this work.

Medium	Components
YPD	Yeast extract, 10 g/L; peptone, 20 g/L; glucose, 20 g/L
SD	Yeast nitrogen base without amino acids, 6.7 g/L; glucose, 20 g/L
SDP	Yeast nitrogen base without amino acids and ammonium sulfate, 1.7 g/L; glucose, 20 g/L; proline, 1 g/L
SDV	Yeast nitrogen base without amino acids and ammonium sulfate, 1.7 g/L; glucose, 20 g/L; valine, 1 g/L
SD+V	SD supplemented with valine, 1 g/L
SD+V+G	SD supplemented with valine, 1 g/L and glycine 1 g/L
SD+γ-EVG	SD supplemented with 100 mg/L of γ-EVG
SD+γ-EV	SD supplemented with 100 mg/L of γ-EV
SD+VG	SD supplemented with 100 mg/L of VG
SDP+VG	SDP supplemented with 100 mg/L of VG

### Analysis of peptide content in yeast cells

The peptide content in the yeast cells was measured by liquid chromatography/tandem mass spectrometry (LC/MS/MS) as described in [62], with some modifications. Five milliliters of the corresponding liquid medium in a 50-ml test tube (d = 20 mm) was inoculated with an overnight culture of the corresponding strain at an optical density (OD_600_) of 0.03–0.2 and cultivated overnight to the late logarithmic phase (OD_600_ = 1.5–3.5). After cultivation, the OD_600_ of the cultures was measured, and the tubes were cooled on ice. Cells from 2 ml of the medium were collected by centrifugation, washed twice with ice water, and resuspended in 1 ml of water. Tubes with cell suspensions were incubated in a water bath at 70°C for 10 min and centrifuged at room temperature for 3 min at 13000 g. The supernatant was collected and filtered with Amicon Ultra– 0.5 ml 10K filter units (Millipore, No UFC501096) at 7000 g, 20 min, 4°C, to remove large peptides. If necessary, before filtration, the water extracts were vacuum-dried and dissolved in a smaller volume to concentrate the peptides. Alternatively, instead of filtration, purification with acetonitrile was carried out. For this purpose, 4 volumes of acetonitrile were added to 1 volume of water extract and mixed; the mixture was incubated at room temperature for 20 min and centrifuged at 13000 g for 20 min at 20°C. Then, the supernatant was collected and vacuum-dried, and the precipitate was dissolved in water. Purified extract solutions were derivatized with an AccQ-Fluor Reagent Kit (Waters, WAT052880) according to the manufacturer’s instructions, but with some modifications: after derivatization, 200 μl of 0.1% aqueous solution of formic acid was added, and the samples were centrifuged at 13000 rpm for 10 min. The resulting supernatant was analyzed with an Agilent 1100 HPLC connected to an API 4000 triple quadrupole mass spectrometer or with an Agilent 1200 HPLC connected to an Agilent 6410 triple quadrupole mass spectrometer equipped with an ESI-Turbo spray (positive mode). The HPLC conditions were as follows: column, Thermo Hypersil-Keystone C18 100 mm*2.1 mm*5 μm or an analogous column; mobile phase A, 0.1% formic acid in water; mobile phase B, 0.1% formic acid in acetonitrile. The ions used for peptide identification by MS/MS analysis are listed in Table 2. The pure peptides γ-EVG, γ-EV and VG were used as standards.

**Table 2:** Ions used for peptide identification by MS/MS analysis.

Peptide	Precursor ion	Product ions
γ-EVG	474	171, 304, 229
γ-EV	417	171, 247, 184
VG	345	171, 270, 242

### Statistical analysis

Statistical analysis was performed using Student’s *t* test. Microsoft Excel 2007 was used for the calculation. The peptide values are presented as arithmetic means of 3–10 independent experiments. The error bars on diagrams represent ± 95% confidence intervals. Additionally, the conclusions regarding the effect of genetic modifications on peptide accumulation were verified using the Mann–Whitney *U* test.

The raw data used to build the graphs are given in S1–S6 Tables.

## Results

### Detection of γ-EVG, γ-EV and VG in yeast cell extracts and dependence of γ-EVG concentration in the extracts on medium composition

**Fig 2:**
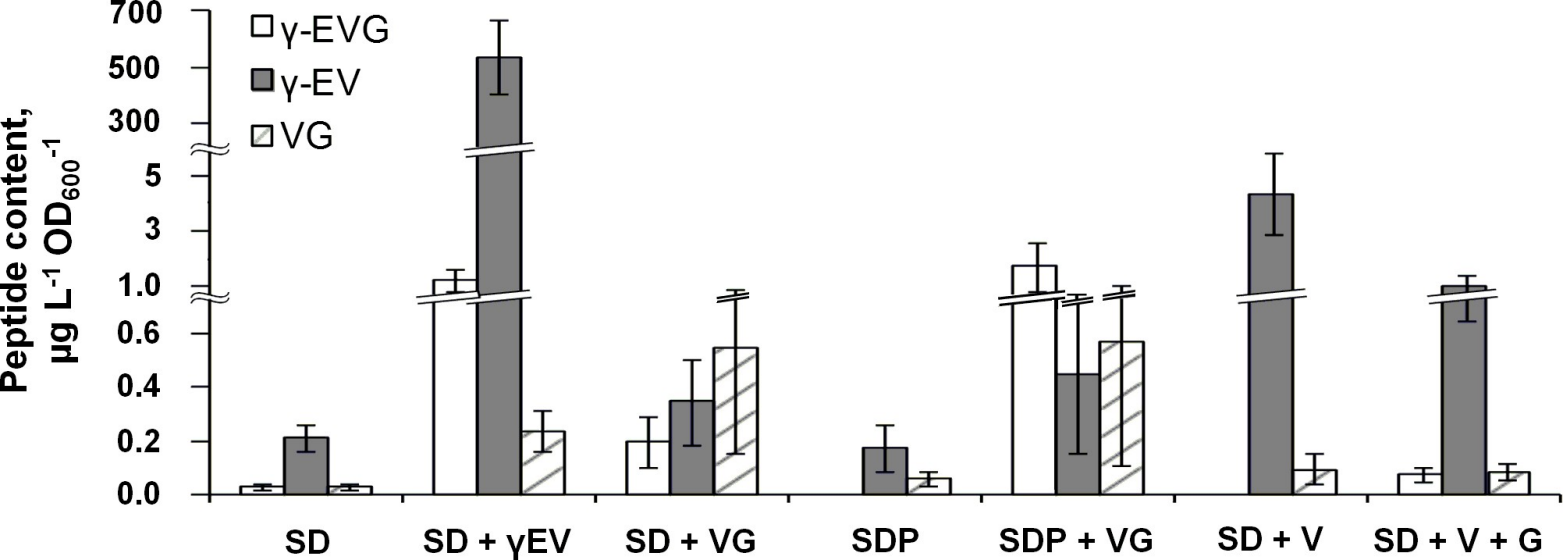
Dependence of peptide concentration in yeast cell extracts on medium composition. Cells of the WT strain were grown in SD or SDP (SD in which ammonium sulfate was replaced with proline as a nitrogen source) medium supplemented with γ-EV, VG, valine, or valine and glycine.

To study the ability of yeast to produce γ-EVG, the peptide content was determined in cells of the WT strain (S288C *ura3*Δ*0*) cultivated in minimal synthetic liquid medium (SD). γ-EVG was detected in these cells at approximately 0.03 μg (L*OD_600_)^-1^ (corresponding to approximately 0.1 μg /g DCW). Two other peptides, namely, γ-EV and VG, were also detected in this extract (Fig 2).

γ-EV and VG could be precursors of γ-EVG in reactions catalyzed by GS and GGT, respectively (Fig 1). To determine which peptide, γ-EV or VG, served as a precursor for γ-EVG synthesis, yeast cells were cultivated in medium supplemented with these peptides, namely, SD+γ-EV and SD+VG, respectively. Cultivation of cells in the SD+γ-EV medium increased the concentrations of both γ-EVG and γ-EV in the cell extracts approximately 50- and 2500-fold, respectively. In cells cultivated in SD+VG, the concentrations of γ-EVG and VG increased approximately 10-fold (Fig 2).

Because ammonia inhibits peptide transport into cells [63], the experiment with VG was repeated in SDP medium (SD medium in which ammonium sulfate was replaced with 1 g/L of proline). Indeed, in cells grown in SDP medium supplemented with VG (SDP+VG), the γ-EVG concentration was approximately 8-fold higher than that in cells grown in SD+VG (Fig 2).

To determine whether amino acids can be precursors of γ-EVG, cells were cultivated in SD medium supplemented with valine (SD+V) or with valine and glycine (SD+V+G). Cultivation in SD+V medium increased the intracellular γ-EV concentration, whereas cultivation in SD+V+G increased the amounts of all three peptides, namely, γ-EV, γ-EVG and VG (Fig 2).

In subsequent experiments, derivatives of three strains with different variants of the *URA3* allele were used: S288C (native *URA3*), S288C *ura3*Δ*0* (deletion of the entire *URA3* coding sequence) and S288C *ura3*Δ*227* (partial deletion of *URA3*). To exclude the influence of the *URA3* allele, the peptide content of these three strains was compared under the conditions described above, and differences between intracellular peptide concentrations were not observed among the strains.

### Identification of the enzyme responsible for the synthesis of γ-EVG from γ-EV

**Fig 3:**
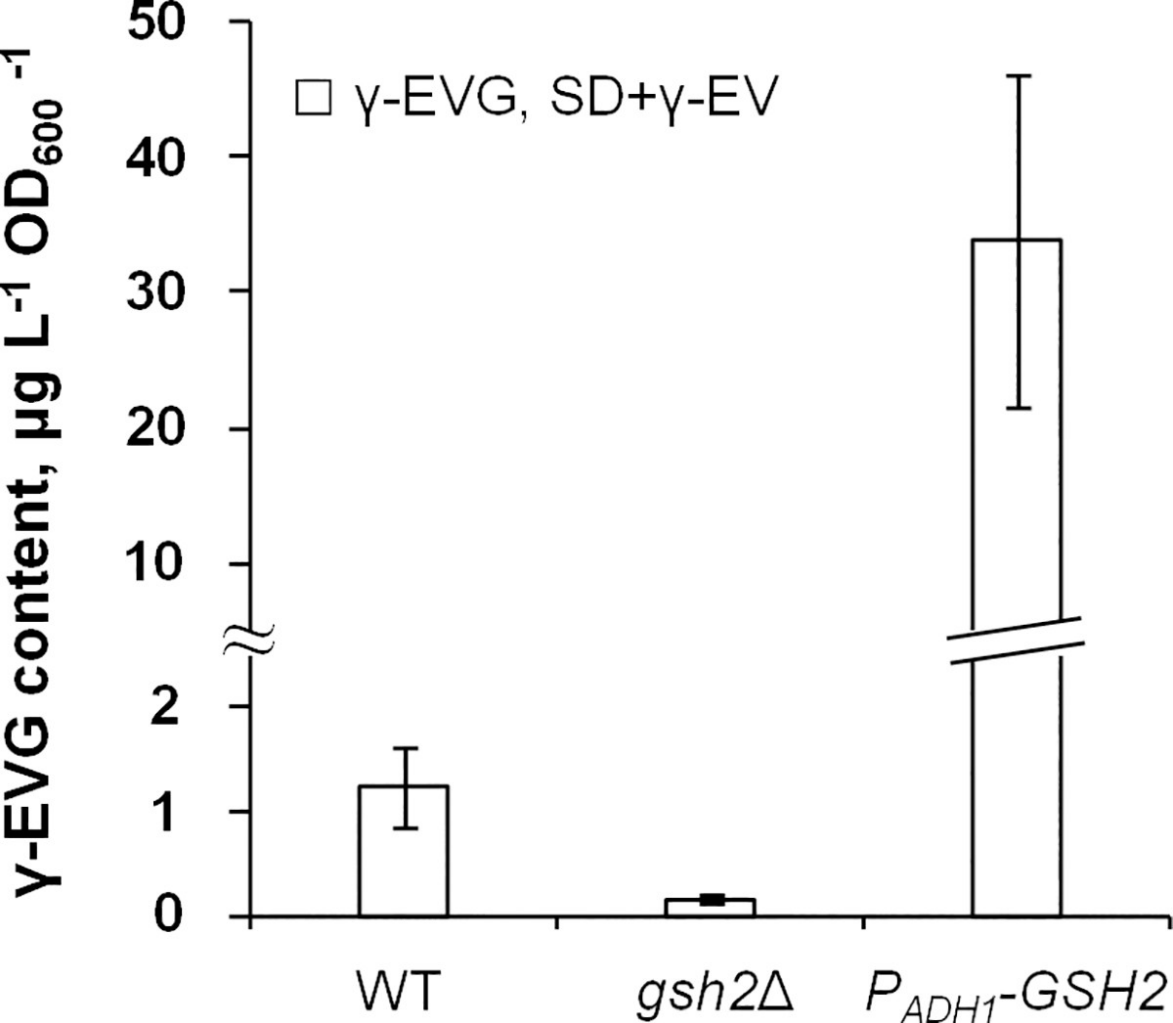
Effect of deletion and overexpression of *GSH2* on the γ-EVG concentrations in cell extracts. Cells were cultivated in SD medium supplemented with γ-EV (SD+γEV). The data shown are the mean values of at least three independent determinations. As a control (WT), the strains S288C and S288C *ura3*Δ*0* were used.

The most likely candidate for catalysis of the conversion of γ-EV to γ-EVG was GS, encoded by *GSH2*. To test this hypothesis, the *GSH2* deletion strain (*gsh2*Δ) and the strain in which the promoter of this gene was substituted with the *ADH1* promoter (overexpression of *GSH2* after promoter replacement was confirmed by qPCR and by measurement of GS activity; S2 File) were cultivated in SD+γ-EV medium, and the γ-EVG content in the cells was measured. The *gsh2*Δ strain accumulated approximately 7-fold less γ-EVG than the strain in which *GSH2* was not deleted. In turn, overexpression of *GSH2* led to an approximately 25-fold increase in γ-EVG concentration in the extract (Fig 3). Thus, these data show that conversion of γ-EV to γ-EVG was catalyzed by Gsh2p.

### Identification of the enzyme responsible for the synthesis of γ-EVG from VG. (1) γ-EVG synthesis was dependent on VG uptake

**Fig 4:**
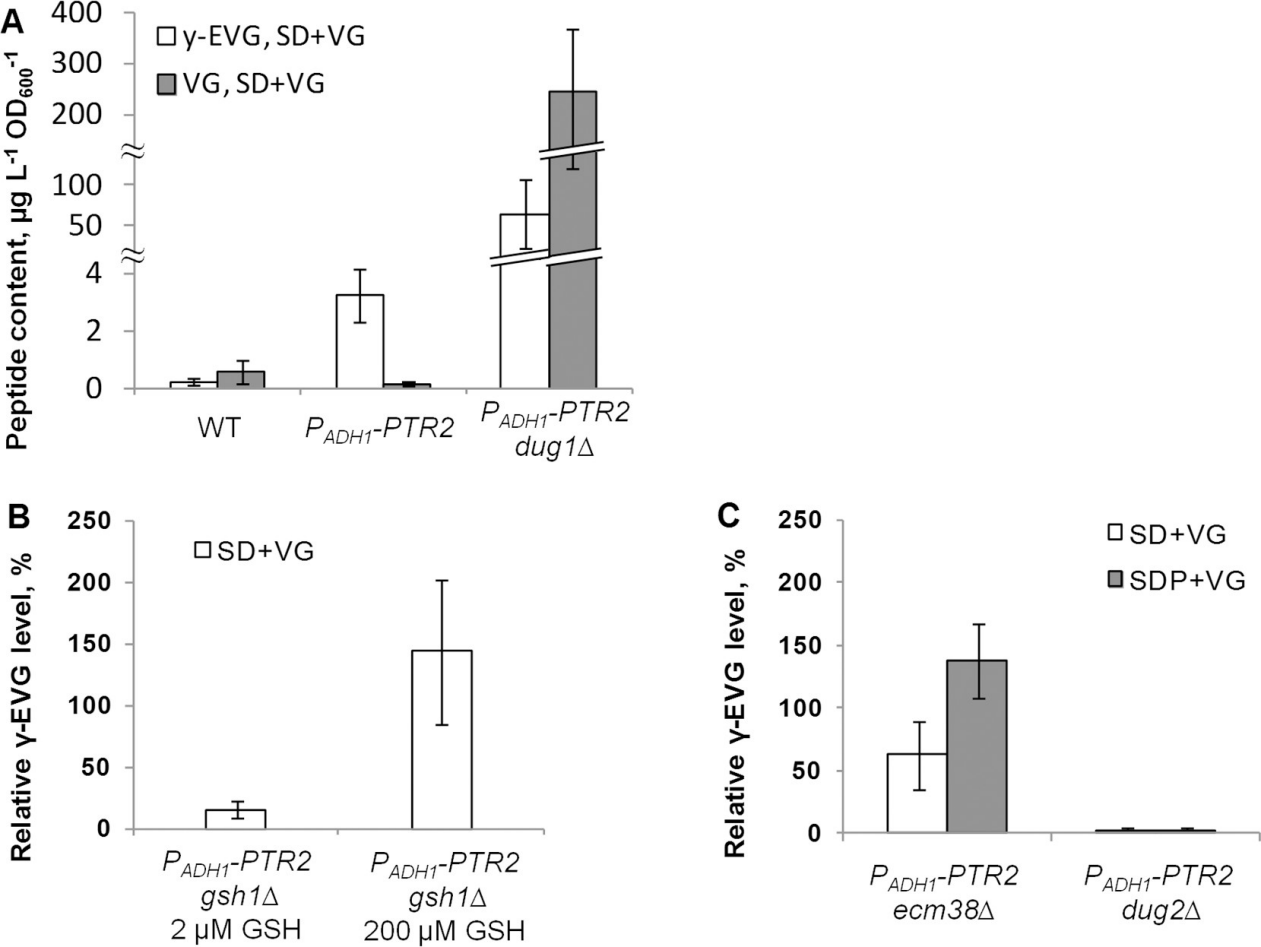
Identification of the enzyme responsible for γ-EVG synthesis from VG. **A, Effect of overexpression of *PTR2* and deletion of *DUG1* on intracellular VG and γ-EVG concentrations.** The strain S288C *ura3*Δ*0* was used as a control (WT). **B. Effect of GSH concentration on γ-EVG synthesis by the *P***_***ADH1***_***-PTR2 gsh1*Δ strain. C. Effect of deletion of *ECM38* or *DUG2* on γ-EVG synthesis by the *P***_***ADH1***_***-PTR2* strain.** In B and C, the level of γ-EVG in the *P*_*ADH1*_*-PTR2* strain cultivated in the corresponding medium was taken to be 100%.

To determine the influence of VG import on the synthesis of γ-EVG, the native promoter of *PTR2*, encoding a di- and tripeptide transporter [64], was replaced by the constitutive promoter *P*_*ADH1*_ in the S288C *ura3*Δ*0* strain. This modification was performed to enhance the import of VG upon cultivation in SD medium supplemented with VG (SD+VG) because it is known that ammonia ions, which are contained in this medium, inhibit uptake of peptides [63]. Cultivation of the resulting and parental strains in SD+VG medium showed that the promoter replacement had no effect on the intracellular VG concentration but resulted in a more than 15-fold increase in the γ-EVG content (Fig 4A).

### Identification of the enzyme responsible for the synthesis of γ-EVG from VG (2). preventing VG degradation increased γ-EVG synthesis

One of the possible reasons why the replacement of the *PTR2* promoter did not result in an increase in the intracellular VG concentration could be a rapid hydrolysis of this peptide in the cells. In this case, it was not clear whether VG itself was a precursor for γ-EVG or whether the precursors were valine and glycine, formed as a result of VG degradation. To confirm the hypothesis of VG degradation, several strains from a yeast deletion collection, bearing deletions of genes encoding peptidases, were cultivated in SDP+VG medium, and the VG concentration was determined in the yeast extracts. Among the tested strains, only the *DUG1* deletion strain demonstrated considerably increased VG content (S3 File). Recently, *DUG1* was identified as a gene encoding the Cys-Gly dipeptidase participating in an alternative pathway of GSH degradation [58]. *DUG1* was deleted in the *P*_*ADH1*_*-PTR2* strain, and the resulting strain was cultivated in SD+VG medium. Determination of the peptide content demonstrated that deletion of *DUG1* increased the VG content in cells more than 1000-fold and the γ-EVG content more than 10-fold (Fig 4A). This result proved the hypothesis that VG itself served as a precursor for γ-EVG synthesis.

### Identification of the enzyme responsible for the synthesis of γ-EVG from VG (3). γ-EVG synthesis was dependent on the GSH supply

To prove that GSH was a γ-glutamyl donor for the synthesis of γ-EVG from VG, GSH-limited conditions were designed. For this purpose, *GSH1*, encoding GCL, the first enzyme in the GSH biosynthetic pathway, was deleted in the *P*_*ADH1*_*-PTR2* strain. Then, the minimal concentration of GSH, leading to growth of the resulting strain in SD at an OD_600_ of approximately 4 units, was determined to be approximately 2 μM. It was also confirmed that the intracellular GSH concentration in cells cultivated in medium supplemented with 2 μM GHS was lower than that in cells cultivated in medium supplemented with 200 μM GSH (S4 File). Then, the *P*_*ADH1*_*-PTR2 gsh1*Δ strain was cultivated in SD+VG medium supplemented with 2 μM or 200 μM GSH. In cells grown in medium supplemented with 2 μM GSH, the concentration of γ-EVG was found to be lower than that in cells cultivated in medium supplemented with 200 μM GSH (Fig 4B). This result supported the hypothesis that GSH served as a γ-glutamyl donor for the synthesis of γ-EVG from VG.

### Identification of the enzyme responsible for the synthesis of γ-EVG from VG (4). γ-EVG synthesis was dependent on the integrity of the (Dug2p-Dug3p)_2_ complex

Based on previous knowledge of degradation of GSH, the first candidate for the transfer of the γ-glutamyl moiety from GSH to VG was GGT, encoded by *ECM38* [54]. To examine this hypothesis, *ECM38* was deleted in the *P*_*ADH1*_-*PTR2* strain. The resulting *P*_*ADH1*_*-PTR2 ecm38*Δ strain accumulated approximately 40% less γ-EVG than the parental strain when cultivated in SD+VG and approximately 40% more when cultivated in SDP+VG media (Fig 4C). Therefore, Ecm38p did not participate in the synthesis of γ-EVG from VG, or its contribution to this process was minor. Thus, it was hypothesized that this reaction could be carried out by the enzymes of a recently discovered alternative GSH degradation pathway [57]. A component of this pathway, namely, the (Dug2p-Dug3p)_2_ complex, has been shown to cleave GSH to glutamate and Cys-Gly [59]. To examine this hypothesis, *DUG2* was deleted in the *P*_*ADH1*_*-PTR2* strain. The resulting *P*_*ADH1*_*-PTR2 dug2*Δ strain accumulated negligible amounts of γ-EVG when cultivated in SD+VG or SDP+VG medium (Fig 4C). Thus, the transfer of the γ-glutamyl moiety from GSH to VG was mainly carried out by the (Dug2p-Dug3p)_2_ complex.

### Identification of the enzyme responsible for γ-EV synthesis

It was assumed that γ-EV can be formed via one of the two pathways, namely, transfer of the γ-glutamyl moiety from GSH to valine (the GGT pathway) or ligation of valine with glutamate by GCL or some other enzyme (Fig 1). It was also supposed that these two pathways can exist simultaneously.

**Fig 5:**
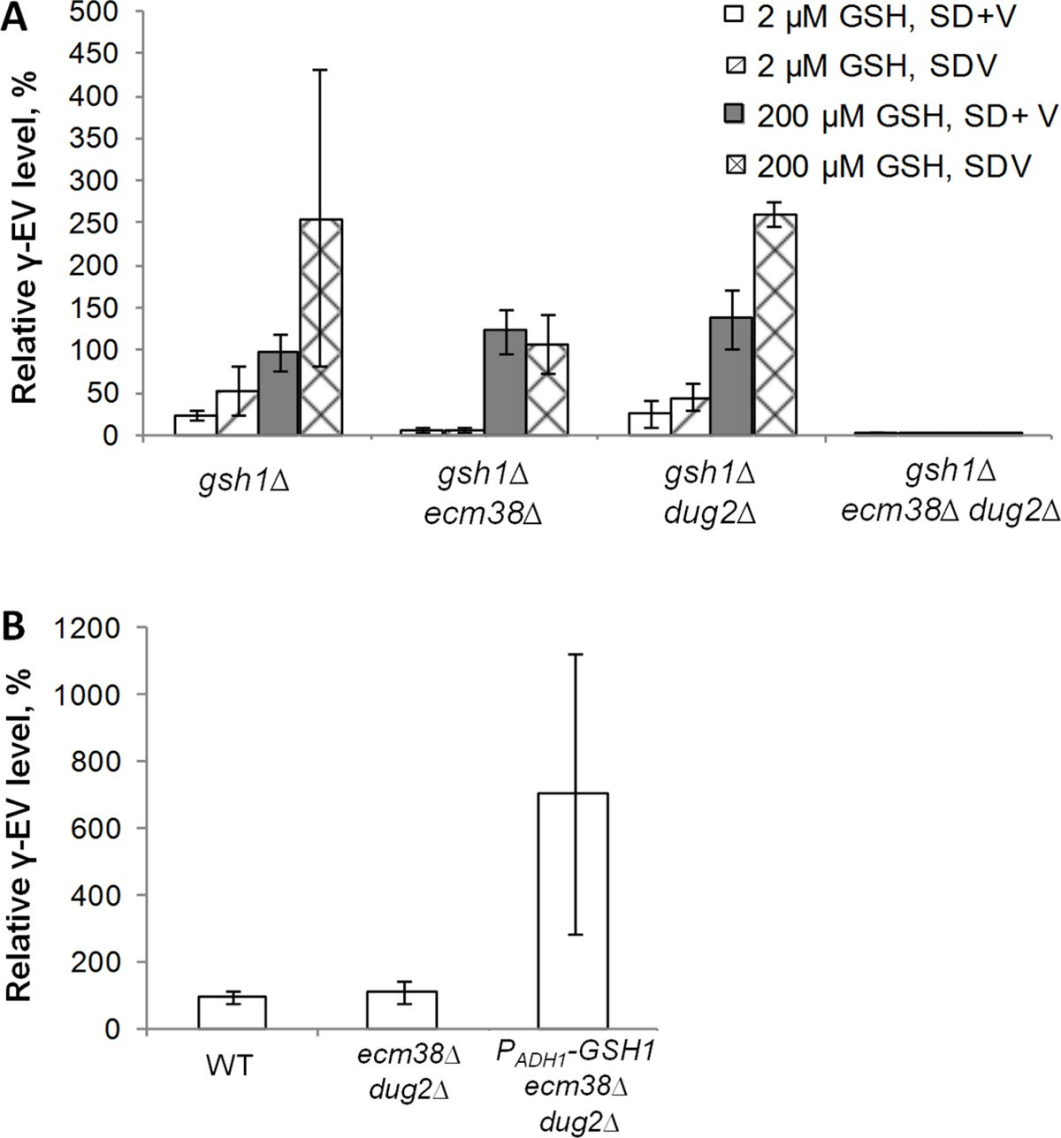
Identification of the enzyme responsible for the synthesis of γ-EV from valine. The level of γ-EV in the WT strain cultivated in SD+V and SDV media was taken to be 100%. **A, Effect of the GSH concentration in the medium and of deletions of *ECM38* and *DUG2* on γ-EV synthesis by the *GSH1* deletion strain. B, Effect of *GSH1* expression on γ-EVG synthesis in the strain with deletions of *ECM38* and *DUG2*.** The strains S288C *ura3*Δ*227* and S288C *ura3*Δ*0* were used as controls (WT).

To evaluate γ-EV synthesis *via* the GGT pathway, a strain with a deletion of *GSH1*, encoding GCL, was used. This strain was cultivated in SD medium supplemented with 1 g/L valine with or without ammonium sulfate (SD+V and SDV, respectively) and limiting or nonlimiting amounts of GSH (2 and 200 μM, respectively). In cells of the *gsh1*Δ strain cultivated in medium supplemented with 2 μM GSH, the γ-EV content was found to be significantly lower than that in the *GSH1* strain cultivated in the corresponding medium without GSH. However, when the concentration of GSH in the medium was 200 μM, the γ-EV content in the *gsh1*Δ strain was restored to a level similar to that in the *GSH1* strain (Fig 5A). Therefore, deletion of *GSH1* did not prevent the synthesis of γ-EV, and the synthesis of this peptide was dependent on the GSH concentration. This result was consistent with the hypothesis that γ-EV is synthesized via the GGT reaction.

To check whether the Ecm38p or (Dug2p-Dug3p)_2_ complex participated in the synthesis of γ-EV from valine and GSH, deletion of *GSH1* was combined with deletions of *ECM38* or *DUG2* and with deletion of both of these genes. The resulting strains were tested for γ-EV accumulation in SD+V and SDV media supplemented with various amounts of GSH. Deletion of neither *ECM38* nor *DUG2* decreased GSH-dependent synthesis of γ-EV. Simultaneous deletion of these two genes decreased the intracellular γ-EV concentration to a barely detectable level (Fig 5A). This residual γ-EV level did not depend on the GSH concentration in the medium. Therefore, both enzymes could participate in the synthesis of γ-EV from valine and GSH, and the activity of thes enzymes was the main source of γ-EV production via this pathway. Notably, the *DUG2* deletion strain accumulated significantly high levels of γ-EV when cultivated in SDV medium supplemented with 200 μM GSH. The intact (Dug2p-Dug3p)_2_ complex likely degraded the surplus γ-EV, which was produced due to derepression of *ECM38* under conditions of nitrogen starvation [65].

Because it was demonstrated that there were no enzymes other than Ecm38p and (Dug2pDug3p)_2_ that produced significant amounts of γ-EV in the *gsh1*Δ strain, a strain with intact *GSH1* and deleted *ECM38* and *DUG2* was used to determine whether γ-EV could be produced by GCL Gsh1p from valine and glutamate. This strain was cultivated in SD+V medium, and the intracellular γ-EV concentration in this strain was found to be similar to that of the WT strain, which harbored intact *ECM38* and *DUG2* (Fig 5B). Considering that the γ-EV level was negligible in the strain *gsh1*Δ *ecm38*Δ *dug2*Δ, it could be concluded that in the *ecm38*Δ *dug2*Δ deletion strain, γ-EV was produced by Gsh1p. To further confirm the ability of Gsh1p to produce γ-EV, the *P*_*ADH1*_*-GSH1 ecm38*Δ *dug2*Δ strain was constructed. Enhancement of *GSH1* transcription after promoter replacement was confirmed by qPCR and by measurement of the GCL activity (S2 File). As expected, this strain produced more γ-EV than the *ecm38*Δ *dug2*Δ strain (Fig 5B).

### Synthesis of γ-EVG by a strain overexpressing *GSH1* and *GSH2*

**Fig 6:**
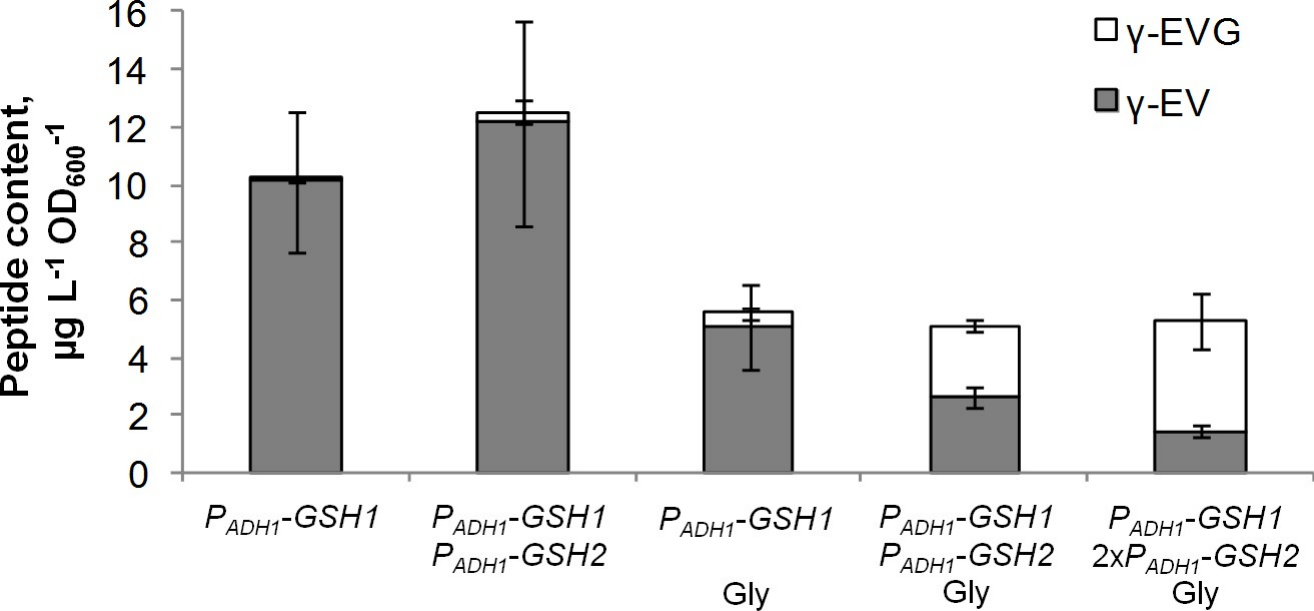
Synthesis of γ-EVG by a strain overexpressing *GSH1* and *GSH2*. All strains were cultivated in SD medium supplemented with valine (SD+V). Glycine was added to the medium where indicated.

Factors influencing the conversion of γ-EV to γ-EVG were studied. The *P*_*ADH1*_*-GSH1* and *P*_*ADH1*_*-GSH1 P*_*ADH1*_*-GSH2* strains were cultivated in SD medium supplemented with valine or with valine and glycine (SD+V or SD+V+G, respectively). Additionally, a derivative of the *P*_*ADH1*_*-GSH1 P*_*ADH1*_*-GSH2* strain containing a second copy of the *P*_*ADH1*_*-GSH2* cassette integrated into the chromosome was cultivated in SD+V+G medium. The *P*_*ADH1*_*-GSH1 P*_*ADH1*_*-GSH2* strain accumulated a significant amount of γ-EVG only when glycine was present in the medium. However, even in the presence of glycine, the *P*_*ADH1*_*-GSH1* strain produced mainly γ-EV and only a small amount of γ-EVG. The strain with two *P*_*ADH1*_*-GSH2* cassettes accumulated more γ-EVG and less γ-EV than the strain with one cassette (Fig 6). These findings indicate that the synthesis of γ-EVG from γ-EV was limited by glycine availability and GS Gsh2p activity.

It is noteworthy that the addition of glycine to the medium decreased γ-EV accumulation by the yeast cells. Apparently, this was due to competitive inhibition of γ-EV synthesis by glycine since for several GCLs, it was shown that this enzyme has a higher affinity for glycine than for valine [39, 40].

### *S*. *cerevisiae* effectively absorbed γ-EVG from the medium, and Opt1p played a main role in this process

**Fig 7:**
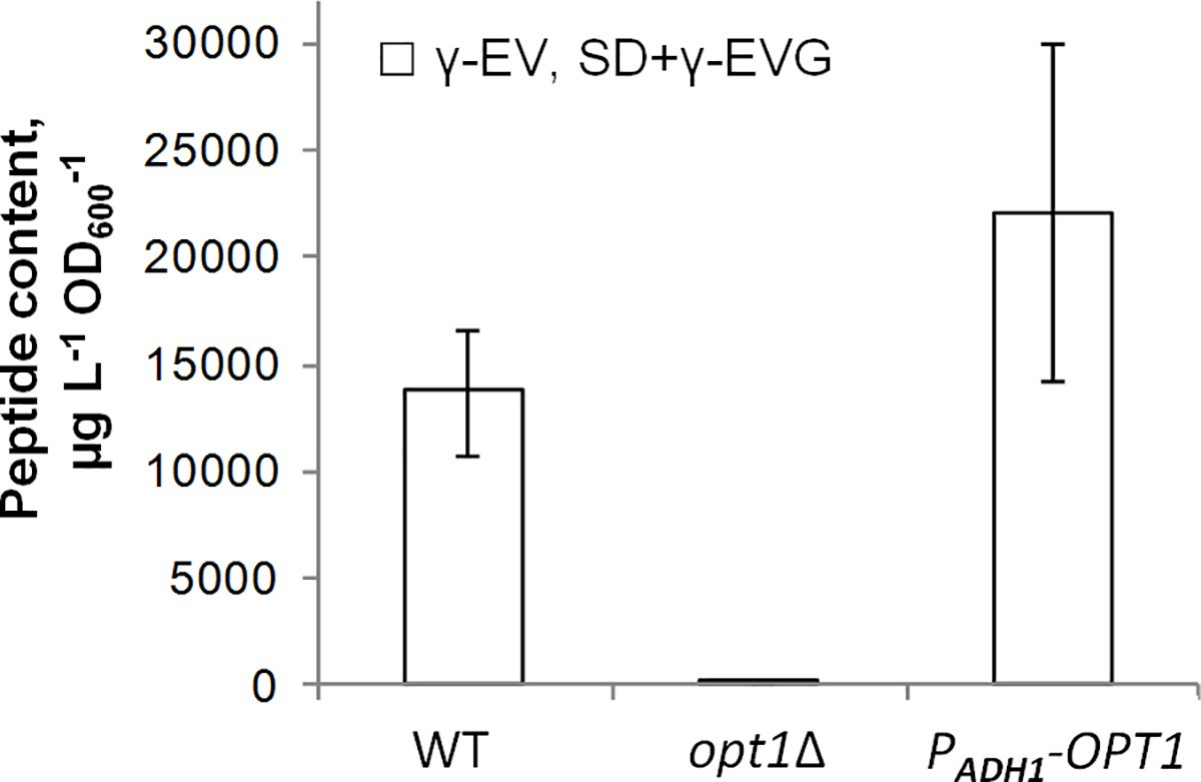
Effect of *OPT1* deletion and overexpression on γ-EVG levels in cells grown in SD+γ-EVG medium. The data shown are the mean values of at least three independent determinations. The strain S288C *ura3*Δ*0* was used as a control (WT).

Because yeast cells absorbed γ-EV with high efficiency from the medium (Fig 2), the uptake of γ-EVG by yeast cells was also studied. It was demonstrated that yeast cells accumulated high amounts of γ-EVG during cultivation in SD medium supplemented with this peptide at 100 mg/L (Fig 7). Deletion of *OPT1* (also called *HGT1* and *GSH11*), encoding a GSH importer [66], was found to considerably decrease the γ-EVG concentration in cells grown in the γ-EVG-containing medium, whereas replacement of the native promoter of this gene with the *ADH1* promoter increased the γ-EVG concentration. Therefore, it may be concluded that Opt1p played a main role in γ-EVG uptake from the medium.

## Discussion

**Fig 8:**
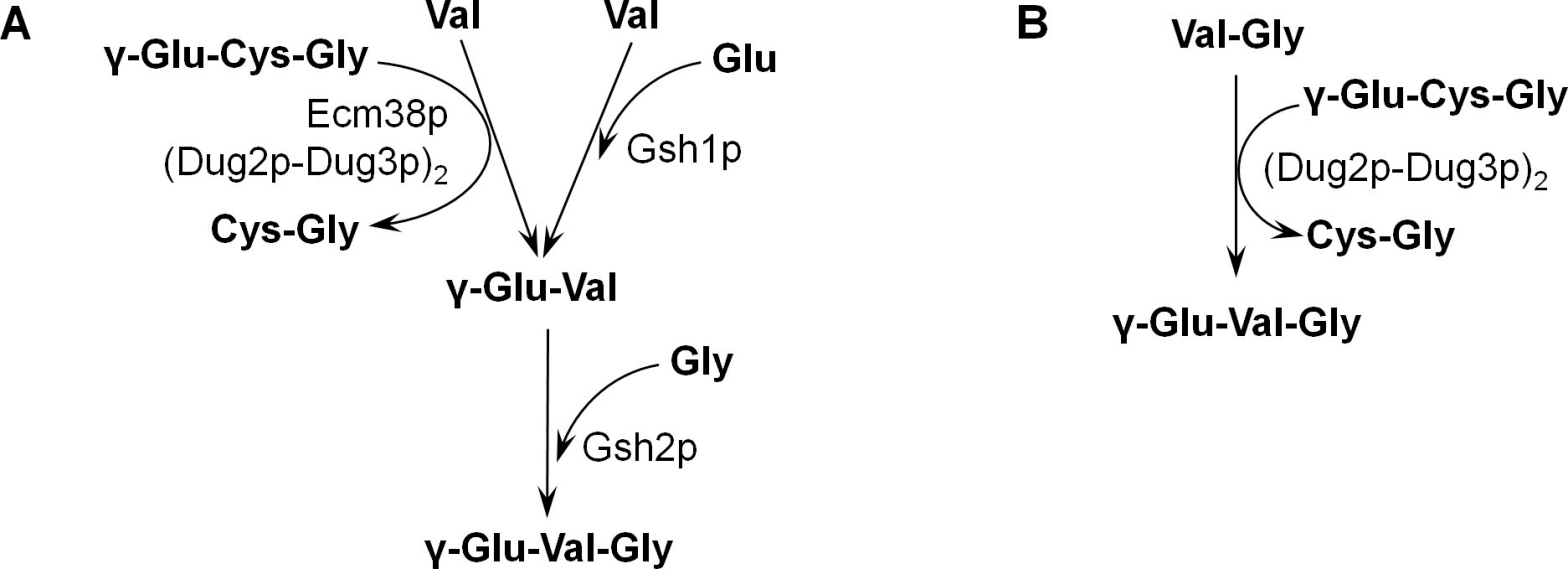
Two pathways of γ-EVG synthesis in *S*. *cerevisiae*. A. Synthesis from valine, glutamate and glycine. B. Synthesis from the dipeptide VG.

In the beginning of this work, two pathways for the synthesis of γ-glutamyl tripeptides in *S*. *cerevisiae* were proposed: (i) synthesis as a side reaction of GSH biosynthesis and (ii) transfer of the γ-glutamyl group from GSH onto other peptides (Fig 1). To prove or refute the existence of these pathways, several experiments were carried out using the tripeptide γ-EVG as a model. *S*. *cerevisiae* was shown to synthesize this peptide, even when the cells were cultivated in minimal synthetic medium. The experiments revealed the presence of both pathways: (i) synthesis by GCL Gsh1p and by GS Gsh2p using glutamate, valine and glycine as precursors; and (ii) synthesis by transfer of the γ-glutamyl residue from GSH to the dipeptide VG. The latter reaction was found to be catalyzed by the (Dug2p-Dug3p)_2_ complex, which was previously identified as part of a fungal-specific alternative GSH degradation pathway [57–59]. It was also demonstrated that in addition to the condensation of valine with glutamate, γ-EV is synthesized via transfer of the γ-glutamyl group from GSH to valine. This reaction was catalyzed by Ecm38p or the complex (Dug2p-Dug3p)_2_. Fig 8 summarizes the data obtained in this work and shows the proposed scheme of γ-EVG synthesis. The contribution of these pathways to the pool of intracellular γ-EVG was dependent on cultivation conditions.

The obtained results, as well as the finding that γ-EV and γ-EVG can be easily imported into yeast cells, indicated that the peptides γ-EV and γ-EVG are integral components of yeast cells. These results also allowed us to propose that γ-glutamyl dipeptides and γ-glutamyl tripeptides, with the structures γ-EX and γ-EXG, respectively, where X is any amino acid, can be produced during normal yeast metabolism.

The results obtained in this work did not allow us to identify the prevalent pathway of γ-EVG formation in wild-type cells cultivated in minimal medium because the γ-EVG level under these conditions was close to the detection limit. However, in the strain overexpressing *GSH1* and *GSH2* cultivated in the medium supplemented with valine and glycine, γ-EVG was clearly produced by Gsh2p from γ-EV and glycine. Most of the γ-EV produced under these conditions was apparently produced by Gsh1p because the γ-GGT reaction is reversible, and in the case of increasing γ-EV concentrations, γ-GGT degraded this peptide (see below).

The results of the present work are also associated with the study of the turnover of GSH and other γ-glutamyl peptides in yeast cells. The GSH concentration in cells is regulated by the synthesis and degradation of this peptide. The half-life of GSH in cells growing in medium supplemented with (NH_4_)_2_SO_4_ as a nitrogen and sulfur source was estimated in various studies as being 990 and 174 min [33, 67], respectively. Additionally, according to Jasper and coauthors, the half-life of GSH was dependent on the nitrogen source [33]. In our experiments, the transfer of the γ-glutamyl group from GSH to valine was found to not be inhibited by ammonium ions (Fig 5.) This finding differed from previously obtained results, in which synthesis of γ-glutamyl derivatives of glutamate and glycine occurred in NH_4_^+^-free medium only [33]. According to our data, both Ecm38p and the (Dug2p-Dug3p)_2_ complex could transfer the γ-glutamyl group from GSH to valine, and deletion of either *ECM38* or *DUG2* did not decrease the synthesis of γ-EV (Fig 5). This indicated that in addition to the synthesis of γ-EV, both enzymes participated in the γ-EV degradation, when the concentration of this peptide exceeds a certain threshold. Therefore, the turnover of other γ-glutamyl peptides, not only GSH, occurred in the yeast cells. Notably, this result also applies to the dipeptide γ-GC, the precursor of GSH. This finding should be considered for further development of the recently constructed strain to produce this compound [68].

The study of the γ-EVG synthesis pathway expanded our knowledge regarding enzymes associated with GSH degradation. To the best of our knowledge, we have demonstrated here for the first time the ability of the (Dug2p-Dug3p)_2_ complex to transfer the γ-glutamyl group from GSH to peptides or amino acids, as well as the ability of Dug1p to digest dipeptide VG.

The effects of γ-EVG on the properties of yeast extract as a food additive obviously depend on the strain and cultivation conditions used. The γ-EVG levels observed in cells grown in minimal synthetic medium was low, but the levels increased when the medium was supplemented with valine and glycine and further increased when *GSH1* and *GSH2* were overexpressed. In WT yeast cells, the regulation of these genes is dependent on several factors, including stress response [69] and the availability of sulfur-containing amino acids [70]. Therefore, during actual fermentation, the expression of *GSH1* and *GSH2* can increase significantly depending on cultivation conditions, and if valine and glycine are present in the medium or are produced because of peptide degradation, the amount of γ-EVG produced can be significant. Synthesis of γ-EVG from the dipeptide VG seems to have no biotechnological value at first glance because of the high price of dipeptides. However, yeast may be cultivated in complex media, which can contain large amounts of peptides, and these peptides can serve as acceptors for the transfer of γ-glutamyl groups from GSH. In this respect, the prevention of VG degradation by inactivation of *DUG1* observed in our study is particularly interesting. It is reasonable to propose that Dug1p may be active toward other dipeptides, and strains with *DUG1* mutations could produce large amounts of γ-glutamyl peptides.

## Supporting information

S1 File Strains construction. (DOCX)

S2 File Effect of replacement of GSH1 and GSH2 promoters. (DOCX)

S3 File Identification of the enzyme responsible for the degradation of VG. (DOCX)

S4 File The influence of GSH concentration on GSH content. (DOCX)

S1 Table Data for Fig 2. (XLSX)

S2 Table Data for Fig 3. (XLSX)

S3 Table Data for Fig 4. (XLSX)

S4 Table Data for Fig 5. (XLSX)

S5 Table Data for Fig 6. (XLSX)

S6 Table Data for Fig 7. (XLSX)
