# Supplementary material for: Multiple pathways for the formation of the γ-glutamyl peptides γ-glutamyl-valine and γ- glutamyl-valyl-glycine in Saccharomyces cerevisiae
Source: PLoS One. 2019 May 9;14(5):e0216622. doi: 10.1371/journal.pone.0216622 (PMC6508711; doi:10.1371/journal.pone.0216622)
Supplement: S1 File — (DOCX) [file pone.0216622.s001.docx]

**Strain construction**

The strains used in this work are listed in Table S1-1. All the strains were derivatives of S288C. All deletions, except for the partial deletion of *URA3* for construction of S288C *ura3*Δ*227*, were created by PCR-mediated gene disruption [1]. For replacement of gene promoters, the method described in [2] was used. The primers used for strain construction are listed in Table S1-2.

**Table S1-1. List of *S. cerevisiae* strains used in this work.**

| **Strain** | **Genotype** | **Source** |
| --- | --- | --- |
| S288C (WT) | *MATa SUC2 mal mel gal2 CUP1 flo1 flo8-1 hap1* | 1 |
| S288C *ura3*Δ*0* (WT) | S288C *ura3*Δ*0*::*P_ADH1_-kanR* | 2 |
| S288C *ura3*Δ*227* (WT) | S288C *ura3*Δ*227::loxP* | 3 |
| *gsh2*Δ | S288C *gsh2*Δ*0*::*P_ADH1_-kanR* | 3 |
| *P_ADH1_-GSH2* | S288C *ura3*Δ*0::P_ADH1_-kanR P_ADH1_-GSH2* | 2 |
| *P_ADH1_-PTR2* | S288C *ura3*Δ*0::P_ADH1_*-*kanR P_ADH1_-PTR2* | 3 |
| *P_ADH1_-PTR2 dug1*Δ | S288C *ura3*Δ*0::P_ADH1_*-*kanR P_ADH1_-PTR2 dug1*Δ0*::loxP-KlURA3-loxP* | 3 |
| *P_ADH1_-PTR2* *gsh1*Δ | S288C *ura3*Δ*0::P_ADH1_*-*kanR P_ADH1_-PTR2 gsh1*Δ*0::loxP-KlURA3-loxP* | 3 |
| *P_ADH1_-PTR2 ecm38*Δ | S288C *ura3*Δ*0::P_ADH1_*-*kanR P_ADH1_-PTR2 ecm38*Δ*0::loxP-KlURA3-loxP* | 3 |
| *P_ADH1_-PTR2 dug2*Δ | S288C *ura3*Δ*0::P_ADH1_*-*kanR P_ADH1_-PTR2 dug2*Δ0*::loxP-KlURA3-loxP* | 3 |
| *gsh1*Δ | S288C *gsh1*Δ*0::P_ADH1_-kanR* | 3 |
| *gsh1*Δ *ecm38*Δ | S288C *ura3*Δ*227::loxP* *ecm38*∆*0::loxP-kanMX-loxP gsh1*∆*0::loxP-KlURA3-loxP* | 3 |
| *gsh1*Δ *dug2*Δ | S288C *ura3*Δ*227::loxP* *dug2*∆*0* *gsh1*∆*0::loxP-kanMX-loxP* | 3 |
| *gsh1*Δ *ecm38*Δ *dug2*Δ | S288C *ura3*Δ*227::loxP* *ecm38*∆*0::loxP-kanMX-loxP gsh1*∆*0::loxP-KlURA3-loxP dug2*∆*0::loxP-bleMX-loxP* | 3 |
| *ecm38*Δ *dug2*Δ | S288C *ura3*Δ*227::loxP* *ecm38*∆*0::loxP-kanMX-loxP* *dug2*∆*0::loxP- KlURA3-loxP* | 3 |
| *P_ADH1_-GSH1 ecm38*∆ *dug2*∆ | S288C *ura3*Δ*0*::*P_ADH1_-kanR* *P_ADH1_-GSH1 dug2*∆*0::loxP-KlURA3-loxP* *ecm38*∆*0::loxP-bleMX-loxP* | 3 |
| *P_ADH1_-GSH1* | S288C *ura3*Δ*0*::*P_ADH1_-kanR* *P_ADH1_-GSH1* | 2 |
| *P_ADH1_-GSH1* *P_ADH1_-GSH2* | S288C *ura3*Δ*0*::*P_ADH1_-kanR* *P_ADH1_-GSH1* *P_ADH1_-GSH2* | 2 |
| *P_ADH1_-GSH1* 2x*P_ADH1_-GSH2* | S288C *ura3*Δ*0*::*P_ADH1_-kanR* *P_ADH1_-GSH1 P_ADH1_-GSH2 pdc1*Δ*0::P_ADH1_-URA3-P_ADH1_-GSH2* | 3 |
| *opt1*Δ | S288C *ura3*Δ*227::loxP* *hgt1*Δ*1981::Agleu2-CaURA3-Agleu2* | 3 |
| *P_ADH1_-OPT1* | S288C *ura3*Δ*0*::*P_ADH1_-kanR P_ADH1_-URA3-P_ADH1_-OPT1* | 3 |

1. Obtained from NITE Biological Resource Center (NBRC).
2. Constructed previously [2].
3. Constructed in this work.

Construction of the S288C *ura3*Δ*0*, *P_ADH1_-GSH1* and *P_ADH1_-GSH1* *P_ADH1_-GSH2* strains was described previously [2]. In that study, these strains were referred to as SOA4, SOA4 *P_ADH1_-GSH1* and SOA4 *P_ADH1_-GSH1* *P_ADH1_-GSH2*, respectively.

To create the strain S288C *ura3*Δ*227::loxP*, contained deletion of 227 nucleotides of *URA3* coding region, the plasmid pKS-URA3-13 [2] was digested with *Stu*I and *Nco*I. This digestion removed 227 nucleotides form the *S. cerevisiae* *URA3* coding sequence, which was contained in 1.6-kb DNA fragment, cloned in this plasmid. After digestion, the *Nco*I site was blunted with the Klenow fragment and the resulting 4.38-kb fragment was ligated with a 1.64-kb *Hinc*II – *Hinc*II fragment from pUG6 (EUROSCARF) [3], containing a *loxP-kanMX-loxP* module. The resulting plasmid was referred to as pKS-URA3-13-kanMX. This plasmid was used as a template for PCR with the primer pair ura3up2 and ura3dn2. The resulting DNA fragment, consisting of the *kanMX* module flanked by *URA3* fragments, was used for transformation of S288C. The resulting transformants contained deletions in *URA3* (*ura3*Δ*227::loxP-kanMX-loxP*). The *kanMX* marker was removed from one of the transformants with the help of a standard procedure using pSH47 (EUROSCARF) [3].

The strain *gsh2*Δ was created by replacement of the *GSH2* coding sequence in S288C with the *P_ADH1_*-*kanR* module. The *P_ADH1_*-*kanR* cassette was produced by PCR using the primer pair GSH2Km-L1 and GSH2Km-R2 and pUC19AOX-G418-BRI [2] as a template. Deletion was confirmed by PCR using the primers GSH2U and GSH2d.

The strain *P_ADH1_-GSH2* was constructed in the same way as the *P_ADH1_-GSH1* *P_ADH1_-GSH2* strain [2], but S288C *ura3*Δ*0* was used as a recipient strain.

To create the strain *P_ADH1_-PTR2*, the *P_AHD1_*-*URA3*-*P_ADH1_* module was produced by PCR using pKS-URA3-PADH1-LR [2] as a template and the primer pair PTR2-PADH1 and PADH1-PTR2. Then the *PTR2* upstream region was amplified from *S. cerevisiae* chromosomal DNA using the primer pair ptr52 and ptr32 and fused with the *P_AHD1_*-*URA3*-*P_ADH1_* module by PCR using the primers ptr52 and PADH1-PTR2. The product of this reaction was used for transformation of the strain S288C *ura3*Δ*0*. Replacement of the *PTR2* promoter was verified using the primer pair ptr52 and ptr2-31.

Disruption of *DUG1*, *GSH1*, *ECM38* and *DUG2* in the SOA4 *P_ADH1_-PTR2* strain was obtained by replacement of the corresponding open reading frames (ORFs) with the *KlURA3* marker. For this purpose, the *loxP-KlURA3-loxP* cassette was amplified by PCR using pUG72 (EUROSCARF) [3] as a template and the following primer pairs: Dug1-pUG72up and Dug1-pUG72d for deletion of *DUG1*; GSH1-UG-up and GSH1-UG-d for deletion of *GSH1*; ECM38-UGup and ECM38-UGd for deletion of *ECM38*; and Dug2-loxPup and Dug2-loxPd for deletion of *DUG2*. Deletions of the genes were confirmed by PCR using the following primers: dug1-51 and dug1-31 for *DUG1*; GSH1U and GSH1d for *GSH1*; ECM38up and ECM38d for *ECM38*; and DUG2up and DUG2d for *DUG2*.

The strain *gsh1*Δ was constructed in the same manner as *gsh2*Δ, but the primer pair GSH1Km-L1 and GSH1Km-R2 was used. Deletion was confirmed by PCR using the primers GSH1U and GSH1d.

The strain *gsh1*Δ *ecm38*Δ was created by sequential disruption of *ECM38* and *GSH1* genes in the strain S288C *ura3*Δ*227::loxP*. The ORF of *ECM38* was replaced by the *loxP-kanMX-loxP* module, which was produced by PCR using pUG6 as a template and the primer pair ECM38-UGup and ECM38-UGd. Disruption was confirmed by PCR using the primers ECM38up and ECM38d. The *GSH1* gene was disrupted using *loxP-KlURA3-loxP* as described above for the strain *P_ADH1_-PTR2* *gsh1*Δ.

The strain *gsh1*Δ *dug2*Δ was created by sequential disruption of the *DUG2* and *GSH1* genes in the strain S288C *ura3*Δ*227::loxP*. For deletion of *DUG2,* the PCR-mediated seamless gene deletion method [4] was applied. *KlURA3* was amplified from pUG72 using the primers DUG2-URA3Kl and Ura3Kl-d.r.-DUG2_tail. The resulting DNA fragment was used as a template for PCR with the primer pair DUG2-URA3Kl and DUG2d-2 to elongate the arm for recombination. The resulting DNA fragment was used for transformation of the strain S288C *ura3*Δ*227::loxP*. The deletion was confirmed by PCR using the primers DUG2up and DUG2d. For deletion of *GSH1,* the *loxP-kanMX- loxP* marker was used. The marker was amplified by PCR using the primer pair GSH1-UG-up and GSH1-UG-d and pUG6 as a template. Deletion was confirmed by PCR using the primers GSH1U and GSH1d.

The strain *gsh1*Δ *ecm38*Δ *dug2*Δ was created by disruption of *DUG2* in strain *gsh1*Δ *ecm38*Δ. The *loxP-bleMX-loxP* cassette was amplified by PCR using pUG66 (EUROSCARF) [3] as a template and the primer pair Dug2-loxPup and Dug2-loxPd. Deletion was confirmed by PCR using the primers DUG2up and DUG2d.

The strain *ecm38*Δ *dug2*Δ was created by sequential disruption of the *ECM38* and *DUG2* genes in the strain S288C *ura3*Δ*227::loxP*. The *ECM38* gene was disrupted using the *loxP-kanMX- loxP* module, and the *DUG2* gene was disrupted using the *loxP-KlURA3-loxP* cassette as described above.

The strain *P_ADH1_-GSH1 ecm38*Δ *dug2*Δ was created by sequential disruption of *DUG2* and *ECM38* in the *P_ADH1_-GSH1* strain. The *DUG2* gene was disrupted using the *loxP-KlURA3-loxP* cassette as described above. *ECM38* was replaced by a *loxP-bleMX-loxP* cassette. For this purpose, this cassette was amplified by PCR using pUG66 as a template and the primer pair ECM38-UGup and ECM38-UGd. Deletion was confirmed by PCR using the primer ECM38d for *ECM38*.

The strain *P_ADH1_-GSH1* 2x*P_ADH1_-GSH2* was constructed by replacement of *PDC1* with the *P_ADH1_*-*URA3*-*P_ADH1_*-*GSH2* cassette in strain *P_ADH1_-GSH1 P_ADH1_-GSH2*. For this purpose, the *P_ADH1_*-*URA3*-*P_ADH1_*-*GSH2* cassette was amplified by PCR using chromosomal DNA from strain *P_ADH1_-GSH1 P_ADH1_-GSH2* as a template and the primer pair PDC1-PADH1 and GSH2-PDC1. The *PDC1* upstream region was amplified by PCR using *S. cerevisiae* chromosomal DNA as a template and the primer pair pdc1-F and PDC1_tail. The resulting DNA fragments were fused by overlap extension PCR using the primer pair pdc1-F and GSH2-PDC1. The resulting DNA fragment was used for transformation of the strain *P_ADH1_-GSH1 P_ADH1_-GSH2*. Integration of the *P_ADH1_*-*URA3*-*P_ADH1_*-*GSH2* cassette into the *PDC1* locus was verified using the primers pdc1-F and pdc1-R.

The strain *opt1*Δ0 was created by disruption of *OPT1* using the *loxP-kanMX-loxP* module. The *loxP-kanMX-loxP* cassette was amplified by PCR using pUG6 as a template and the primer pair hgt1-pUG6u and hgt1-pUG6d. Disruption was confirmed by PCR using the primers hgt1-51 and hgt1-31.

The strain *P_ADH1_-OPT1* was produced in the same way as *P_ADH1_-PTR2*, but the primers HGT1-PADH1 and PADH1-HGT1 were used for amplification of the *P_AHD1_*-*URA3*-*P_ADH1_* module; the primers hgt53 and hgt33 were used for amplification of the *OPT1* upstream region; the primers hgt53 and PADH1-HGT1 were used for generation of the DNA fragment for transformation of the recipient strain. Replacement of the *OPT1* promoter was confirmed using the primers hgt1-51 and hgt1-31.

**Table S1-2. List of primers used in this work.** Hyphens separate the part of each primer that anneals to the template from the arm used for recombination.

| **Primer** | **Sequence (5 '→3')** |  |
| --- | --- | --- |
| ura3up2 | gaggctactgcgccaatt |  |
| ura3d2 | gaagtcattgacacagtctgtga |  |
| GSH2Km-L1 | atggcacactatccaccttccaaggatcaattgaatgaat-ccatccttttgttgtttccgggtgta |  |
| GSH2Km-R2 | ctagtaaagaataatactgtccaaacatccgaatcctgcc-tcagaagaactcgtcaagaaggcgat |  |
| GSH2U | taaatgtctcccaaatcaac |  |
| GSH2d | agcaaatttatgtagaagtgt |  |
| PTR2-PADH1 | aaggtataccacagtgctgctccactgcaa-atttcggatatccttttgttgtttc |  |
| PADH1-PTR2 | cctgagcatcatctgagccttggctgggatggttgagcat-tgtatatgagatagttgattgtatgc |  |
| ptr52 | ggctgcttctcccctttca |  |
| ptr32 | ttgcagtggagcagcact |  |
| ptr2-31 | cccgtcaacgtaatatgg |  |
| Dug1-pUG72up | gaatctcccttcattgttagttcagttgctccagagcatcgtacacaagc-cagctgaagcttcgtacgc |  |
| Dug1-pUG72d | cttgaatgacatggaatttacacatatatacacatatataaataagatgac-gcataggccactagtggatctg |  |
| GSH1-UG-up | acattgtagggtggtttagagtatcgaaaatatacatatagaagaataaa-cagctgaagcttcgtacgc |  |
| GSH1-UG-d | cccaaatcgataatgtcaactttctttcacaaccgaagtaaaaggagt-gcataggccactagtggatctg |  |
| ECM38-UGup | aaaaagacaagacaaaaagtagcgtactagactta-cagttcgctgaagcttcgtacgc |  |
| ECM38-UGd | tatcttgaactatattacagtaccattcttaccccacatc-gcataggccactagtggatctg |  |
| Dug2-loxPup | cgaacaaccctgtaaggaaaagtgaaaaacgagggcagaagtaattgtgaaatc-gtcgacaacccttaatataac |  |
| Dug2-loxPd | acatatgcaaattgggtatatattaagcactttaaaatcaattgtttgtagttgt-ctagtggatctgatatcacc |  |
| dug1-51 | cggctacaagaaaatgtgtcaaaa |  |
| dug1-31 | agttttctgtttttcctctcttttcaga |  |
| GSH1U | tctcgagtcacttgtagaagctg |  |
| GSH1d | aataaatggtcacgttggcat |  |
| ECM38up | gagggaacataacggcatt |  |
| ECM38d | ctgcaaggtagggaaactt |  |
| DUG2up | tgctgcaattagaagttcaatg |  |
| DUG2d | ggttggagcttttatacgcctt |  |
| DUG2-URA3Kl | aggaaaagtgaaaaacgagggcagaagtaattgtgaaatc-cgagagctcgttttatttag |  |
| Ura3Kl-d.r.-DUG2_tail | caattgtttgtagttgt-taattcagttacacacattttattgatattggtgccacag-acgtgatcttttgtaa |  |
| DUG2d-2 | attaggtagaggcctacatatgcaaattgggtatatattaagcactttaaaat-caattgtttgtagttgt |  |
| PDC1-PADH1 | ttgcataatattgtccgctg-atttcggatatccttttgtt |  |
| GSH2-PDC1 | tacataaaaatgcttataaaactttaactaataattagagattaaatcgc-ctagtaaagaataatactgt |  |
| pdc1-F | caagcaaggcagaaactaac |  |
| PDC1_tail | cagcggacaatattatgcaa |  |
| pdc1-R | cttggttccactaattcatc |  |
| hgt1-pUG6u | ggaaagccaaaaattcgacaggcattccatacaggaggaaggtctcg-cagctgaagcttcgtacgc |  |
| hgt1-pUG6d | ttgccacacctgcttcaacaccggcccccatgacgaaattgtac-gcataggccactagtggatctg |  |
| hgt1-51 | ttaatcacatcaaacccactgc |  |
| hgt1-31 | gctaaatattgcaggggtcg |  |
| PADH1-HGT1 | ccgactccaacgagtcgctctccctataaatggtactcat-tgtatatgagatagttgattgtatgc |  |
| HGT1-PADH1 | attcaatgaattgtttcgactatatatggacaatcatcgg-atttcggatatccttttgttgtttc | |
| hgt53 | gatcgatactaacttccgagac |  |
| hgt33 | ccgatgattgtccatatatagt |  |

**SUPPLEMENTAL REFERENCES**

1. Baudin A, Ozier-Kalogeropoulos O, Denouel A, Lacroute F, Cullin C. A simple and efficient method for direct gene deletion in *Saccharomyces cerevisiae*. Nucleic Acids Research. 1993;21(14): 3329-3330.
2. Sofyanovich OA, Nishiuchi H, Yamagishi K, Maekawa K, Serebryanyy VA. A new method for repeated "self-cloning" promoter replacement in *Saccharomyces cerevisiae*. Mol Biotechnol. 2011;48(3): 218-27.
3. Güldener U, Heck S, Fiedler T, Beinhauer J, Hegemann JH. A new efficient gene disruption cassette for repeated use in budding yeast. Nucleic Acids Research. 1996;24: 2519-2524.
4. Akada R, Kitagawa T, Kaneko S, Toyonaga D, Ito S, Kakihara Y, et al. PCR-mediated seamless gene deletion and marker recycling in *Saccharomyces cerevisiae*. Yeast. 2006;23: 399–405.
