# Supplementary material for: Multiple pathways for the formation of the γ-glutamyl peptides γ-glutamyl-valine and γ- glutamyl-valyl-glycine in Saccharomyces cerevisiae
Source: PLoS One. 2019 May 9;14(5):e0216622. doi: 10.1371/journal.pone.0216622 (PMC6508711; doi:10.1371/journal.pone.0216622)
Supplement: S2 File — (DOCX) [file pone.0216622.s002.docx]

**Effect of replacement of native promoters of *GSH1* and *GSH2* on the transcription level of these genes and on the corresponding enzymatic activities.** Overexpression of *GSH1* and *GSH2* after promoter replacement was confirmed by RT-qPCR and by measurement of corresponding enzymatic activities (Table S2).

**Table S2. Changing in transcription of *GSH1* and *GSH2* and in GCL and GS activities after substitution of the *GSH1* and *GSH2* promoters**

| Strain | Relative *GSH1* mRNA concentration | Relative GCL activity | Relative *GSH2* mRNA concentration | Relative GS activity |
| --- | --- | --- | --- | --- |
| SOA4 | 1 | 1 | 1 | 1 |
| SOA4 *P_ADH1_-GSH1* | 11 ± 1 | 3.3 | - | - |
| SOA4 *P_ADH1_-GSH2* | - | - | 7 ± 1 | 14 |

The qPCR results are mean values of three independent determinations. The standard deviation is given as an error. The enzymatic activities are the result of a single measurement.

For each strain, Rt-qPCR analysis was done for three independently grown cultures. Total RNA was isolated according to protocol described in [1]. Reverse transcription was carried out using RevertAid H Minus First Strand cDNA Synthesis Kit, K1631, Thermo Fisher Scientific, and oligo(dT)_18_ primer. qPCR analysis was performed on ANK32 thermocycler (Syntol, Moscow, Russia) using 2.5x SYBR Green qPCR kit M-423, Syntol, Moscow, Russia.

GCL (Gsh1p) activity was measured using crude cell extracts according to the procedure described in [2], with some the modifications. The enzyme activity was assessed by the amount of γ-GC produced. The reaction volume was reduced to 200 µl. Reaction was stopped with addition of 100 µl of ethanol and proteins were removed by centrifugation. Supernatant was diluted 10 times with water, and γ-GC content was determined by HPLC according to the procedure described in [3].

GS (Gsh2p) activity was determined similarly to the GCL activity, but γ-GC and glycine were used as substrates. The enzyme activity was assessed by the amount of GSH produced. The amount of GSH was also determined by HPLC according to the procedure described in [3].

**References**

1. Collart MA, and Oliviero S. Preparation of Yeast RNA. In Ausubel FM, Brent R, Kingston RE, Moore DD, Seidman JG, Smith JA, Struhl K, editors. Current Protocols in Molecular Biology. New York: Wiley; 1993. pp. 13.12.1-13.12.5
2. Kistler M, Maier K, Eckardt-Schupp F. Genetic and biochemical analysis of glutathione-deficient mutants of *Saccharomyces cerevisiae*. Mutagenesis. 1990;5: 39-44.
3. Nishiuchi H, Suehiro M, Sugimoto R, Yamagishi K. Preparation of a γ-glutamylcysteine-enriched yeast extract from a newly developed GSH2-deficient strain. Biosci Bioeng. 2013;115(1): 50-4. doi: 10.1016/j.jbiosc.2012.08.009.
