# Supplementary material for: Multiple pathways for the formation of the γ-glutamyl peptides γ-glutamyl-valine and γ- glutamyl-valyl-glycine in Saccharomyces cerevisiae
Source: PLoS One. 2019 May 9;14(5):e0216622. doi: 10.1371/journal.pone.0216622 (PMC6508711; doi:10.1371/journal.pone.0216622)
Supplement: S3 File — (DOCX) [file pone.0216622.s003.docx]

**Identification of the enzyme responsible for the degradation of VG.** Derivatives of BY4741from a yeast deletion collection, bearing deletions of genes encoding peptidases, were cultivated in SDP+VG medium supplemented with His, 20 mg/L; Leu, 60 mg/L; Met, 20 mg/L and uracil, 20 mg/L; and the VG concentration was determined in the yeast extracts (Table S3).

**Table S3.** **The influence of the genes, encoding peptidases, on the intracellular VG concentration**

| Deleted gene | | OD_600_ | VG,  μg l^-1^ OD_600_^-1^ |
| --- | --- | --- | --- |
| Systematic ORF Name | Standard Gene Name |  |  |
| BY4741 (control) | | 2.5 | 0.02 |
| YHR047C | *AAP1* | 2.2 | 0.02 |
| YHR028C | *DAP2* | 2.1 | 0.02 |
| YHR132C | *ECM14* | 2.1 | 0.07 |
| YKR087C | *OMA1* | 2.2 | 0.02 |
| YFR044C | *DUG1* | 2.5 | 459 |
| YOL098C | *SDD3* | 2.1 | 0.02 |
| YMR089 | *YTA12* | 1.5 | 0.13 |
| YKL103C | *LAP4* | 1.9 | 0.05 |
| YMR297W | *PRC1* | 2.1 | 0.05 |
| YNL239W | *LAP3* | 2.3 | 0.02 |
| YNL191W | *DUG3* | 2.3 | 0.02 |
| YPL154C | *PEP4* | 1.9 | 0.03 |
| YBL022C | *PIM1* | 2.1 | 0.02 |
| YEL060C | *PRB1* | 2.1 | 0.02 |
| YDR430C | *CYM1* | 2.2 | 0.05 |
| YGL203C | *KEX1* | 2.2 | 0.05 |
| YBR286W | *APE3* | 2.0 | 0.05 |
| YBR281C | *DUG2* | 1.9 | 0.05 |
| YCL038C | *ATG22* | 2.1 | 0.05 |
| YCR068W | *ATG15* | 1.8 | 0.06 |
| YPR024W | *YME1* | 2.1 | 0.07 |
| YBR128C | *ATG14* | 2.5 | 0.02 |
| YBR139W | *ATG42* | 2.3 | 0.02 |
