# Supplementary material for: Multiple pathways for the formation of the γ-glutamyl peptides γ-glutamyl-valine and γ- glutamyl-valyl-glycine in Saccharomyces cerevisiae
Source: PLoS One. 2019 May 9;14(5):e0216622. doi: 10.1371/journal.pone.0216622 (PMC6508711; doi:10.1371/journal.pone.0216622)
Supplement: S4 File — (DOCX) [file pone.0216622.s004.docx]

**The influence of GSH concentration in the medium on GSH content in the *P_ADH1_*-*PTR2* *gsh1*∆ strain.** Tubes with 2 ml of SD medium supplemented with 2 or 200 µM GSH and 20 mg/L uracil were inoculated with 1µL of an overnight culture of the *P_ADH1_*-*PTR2* *gsh1*∆ strain, grown in SD medium supplemented with 2 µM GSH and 20 mg/L uracil. Tubes were incubated for 24 hours at 37 °C on rotary shaker at 240 rpm. After cultivation, the optical density of cultures was measured and 1 ml of culture was taken for GSH content analysis. Extraction of GSH was done as described for the peptides extraction in this publication. The GSH content was measured by HPLC as described in [1]. The result is shown in Table S4.

**Table S4.** **The influence of GSH concentration in the medium on GSH content in the *P_ADH1_*-*PTR2* *gsh1*∆ strain.**

| GSH in the medium, µM | GSH in cells,  mg l^-1^ OD_600_^-1^ |
| --- | --- |
| 2 | < 0.1 |
| 200 | 0.56 ± 0.02 |

**References**

1. Nishiuchi H, Suehiro M, Sugimoto R, Yamagishi K. Preparation of a γ-glutamylcysteine-enriched yeast extract from a newly developed GSH2-deficient strain. Biosci Bioeng. 2013;115(1): 50-4. doi: 10.1016/j.jbiosc.2012.08.009.
